# Secoyanhusamine A, an Oxidatively Ring-Opened Isoquinoline Inner Salt From *Corydalis yanhusuo*

**DOI:** 10.3389/fchem.2021.831173

**Published:** 2022-02-01

**Authors:** Lingyan Wang, Huan Xia, Yuzhuo Wu, Yanan Wang, Pengcheng Lin, Sheng Lin

**Affiliations:** ^1^ Key Laboratory of Chinese Internal Medicine of Ministry of Education and Beijing, Dongzhimen Hospital, Beijing University of Chinese Medicine, Beijing, China; ^2^ State Key Laboratory of Bioactive Substance and Function of Natural Medicines, Institute of Materia Medica, Chinese Academy of Medical Sciences and Peking Union Medical College, Beijing, China; ^3^ College of Pharmaceutical Sciences, Qinghai University for Nationalities, Xining, China

**Keywords:** *Corydalis yanhusuo*, *seco*-isoquinoline alkaloid, secoyanhusamine A, acetylcholinesterase inhibitor, Alzheimer’s disease

## Abstract

Secoyanhusamine A (**1**), a rare rearranged *seco*-isoquinoline alkaloid derived from ring oxidative cleavage, was isolated from an aqueous extract of *Corydalis yanhusuo* tubers, together with its biosynthetic precursor dehydrocorybulbine (**2**). Secoyanhusamine A (**1**) was the first example of a highly oxidized isoquinoline inner salt resulting in a 5-(2-azanylethyl)-2-carboxylate-4-oxo-4*H*-pyran ring system. The biosynthetic pathway of **1** was also postulated. Secoyanhusamine A (**1**) exhibited potent inhibition against acetylcholinesterase (AChE) with an IC_50_ value of 0.81 ± 0.13 *μ*M. Molecular simulation docking demonstrated that **1** created a strong interaction with the Asp-74 residue of AChE *via* attractive charge of the quaternary nitrogen.

## Introduction

Acetylcholinesterase (AChE) has been regarded as an attractive target in the treatment of Alzheimer’s disease (AD) (Amat-ur-Rasool et al., 2021), the most common neurodegenerative disease in old age that is characterized clinically by progressive memory loss, cognitive dysfunction, language disorders, and personality changes (Dong et al., 2012; Kepp, 2012). The maintenance of acetylcholine (ACh) levels *via* inhibition of AChE has shown to be an effective therapy in the amelioration of the AD symptoms. Indeed, several drugs such as donepezil, galantamine, and rivastigmine have been approved defined upon this approach (Tumiatti et al., 2008).

Previous studies have shown that natural products are the great resources of candidate drugs for the treatment of AD (Irajia et al., 2020; Tang et al., 2021). One of the interesting resources of potential active compounds, such as isoquinoline alkaloids, is the plant of the Papaveraceae family (Adsersen et al., 2007; Hu et al., 2009; Hung et al., 2010; Zhou et al., 2012; Hostalkova et al., 2019; Zhang et al., 2019). The most studied isoquinoline alkaloid is berberine, which could improve cognitive impairment by promoting autophagic clearance in the mouse model of AD (Wu et al., 2021; Ye et al., 2021). In addition, other isoquinoline alkaloids with AChE inhibitory activity have the potential to act against AD, for instance, palmatine, jatrorrhizine, and coptisine (Ingkaninan et al., 2006; Jung et al., 2009; Hung et al., 2010; Tsai and Lee, 2010; Xiao et al., 2011; Brunhofer et al., 2012; Huang et al., 2012; Mollataghi et al., 2012; Cao et al., 2018; Liu et al., 2019).

**FIGURE 1:**
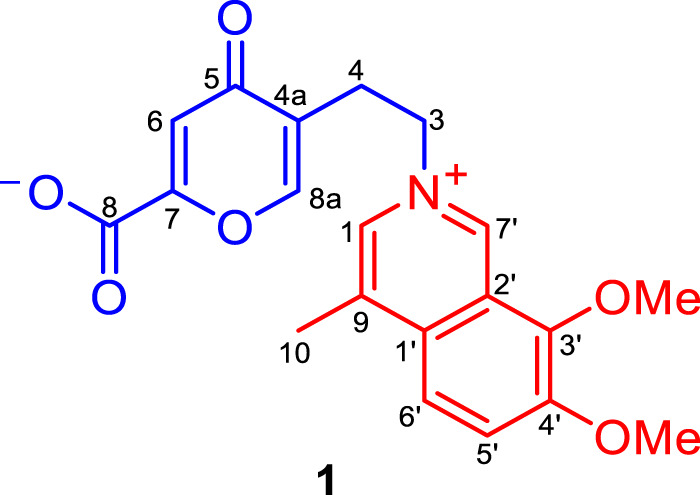
Structure of **1**.

The dried tuber of *Corydalis yanhusuo* W.T. Wang (Papaveraceae), also known as “Yuan-Hu”, is an important traditional Chinese medicine for the treatment of spasms and menstrual and abdominal pain (He et al., 2007; Chinese Pharmacopoeia, 2020). Recent study illustrated that a Chinese medicine prescription Yuan-Hu Zhi Tong (YZT) comprising of *C. yanhusuo* tubers could alleviate the AD symptoms in the P301S tau and 3XTg-AD mice model, of which the major bioactive constituents are the isoquinoline alkaloids originated from *C. yanhusuo* (Iyaswamy et al., 2020). In addition, during our preliminary AChE inhibition activity screening, the YH-D fraction of the *C. yanhusuo* tubers aqueous extract was hit, with an IC_50_ value of 8.16 ± 1.06 *μ*g/ml. Inspired by this finding, a bioactivity-guided isolation strategy was used to explore promising AChE inhibitors from this bioactive fraction, which resulted in the isolation of a rare rearranged *seco*-isoquinoline alkaloid with potent AChE inhibition, named secoyanhusamine A (**1**) (Figure 1), together with a biosynthetic precursor dehydrocorybulbine (**2**). We report details of the isolation, structure elucidation, possible biogenetic pathway, and AChE inhibitory activity of **1**.

## Materials and Methods

### General Experimental Procedures

The UV spectrum was acquired on a Cary 300 spectrometer. The IR spectrum was obtained on a Nicolet Impact 400 FT-IR spectrophotometer. 1D- and 2D-NMR spectra were recorded using Bruker 600 MHz spectrometers (600 MHz for ^1^H and 150 MHz for ^13^C), and the chemical shifts were reported as *δ* values using internal standard TMS (measured in MeOH-*d*_4_). HR-ESIMS data were obtained on Agilent 1100 Series LC-MSD-Trap-SL and Agilent 6520 Accurate-Mass Q-TOFL CMS spectrometers (Agilent Technologies, Ltd., Santa Clara, CA, United States). Column chromatography (CC) was performed with a macroporous resin (HP20, Mitsubishi Group, Japan), Sephadex LH-20 (Amersham Biosciences, Sweden), and silica gel (200–300 mesh, Qingdao Marine Chemical Inc., People’s Republic of China). Analytical HPLC was performed with an Agilent 1260Ⅱ using a Titank column (Guangzhou FLM Scientific Instrument Co., Ltd.) packed with C_18_ (250 × 4.6 mm, 5 *μ*m). HPLC separation was performed on a system consisting of a Waters 600 controller, a Waters 600 pump, and a Waters 2487 Dual *λ* absorbance detector (Waters Corporation, Milford, MA, United States), with a Shiseido MGII ODS C_18_ column (250 × 10 mm or 250 × 20 mm, 5 *μ*m). TLC was conducted on precoated silica gel GF_254_ plates. Spots were visualized under UV light (254 or 356 nm) or by spraying with 10% H_2_SO_4_ in 95% EtOH followed by heating or with a Dragendorff’s reagent. All chemicals were obtained from commercially available sources and were used without further purification.

### Plant Material

See ref. Wang et al., 2020.

### Extraction and Isolation

For extraction and preliminary fractionation of the extract, see ref. Wang et al., 2020. The 50% EtOH fraction (YH-D) was evaporated under reduced pressure to yield 270 g of a residue, which was subjected to column chromatography (CC) on MCI, with H_2_O (40 L), 30% EtOH (100 L), 60% EtOH (100 L), and 95% EtOH (100 L) as successive eluents to yield four major fractions. The 30% EtOH fraction was chromatographed by MPLC over a reversed-phase C_18_ silica gel eluting with MeOH–H_2_O (0–95%), to afford five fractions (A−E). Fraction A (19.54 g) was subsequently fractionated by Sephadex LH-20 CC eluting with 10% MeOH–H_2_O to furnish six subfractions (A1−A6). Subfraction A4 was separated further by ODS C_18_ MPLC eluting with MeOH–H_2_O (0–50%), to afford seven fractions (A4-1−A4-7). Subfraction A4-6 was separated further by semipreparative HPLC [RP_18_, 5 *μ*m, 250 × 10 mm, 254 nm, CH_3_CN−H_2_O−TFA (25:75:0.1)] to give **1** (*t*_R_ = 12.7 min, 2.0 mg) and **2** (*t*_R_ = 17.5 min, 5.0 mg). The 95% EtOH fraction (YH-E) was subjected to a silica gel CC, eluting with CH_2_Cl_2_−MeOH (200:1 → 10:1), to afford nine fractions (A−I). Fraction I was fractionated by Sephadex LH-20 CC eluting with MeOH to yield eight subfractions (I1−I8). Subfraction I7 was separated further by preparative HPLC to give **2** [RP_18_, 5 *μ*m, 250 × 20 mm, 280 nm, MeOH−H_2_O−TFA (52:48:0.1), *t*_R_ = 21.3 min, 250 mg].

### Compound Characterization

*Secoyanhusamine A* (**1**): a yellow, amorphous powder; UV (MeOH) *λ*_max_ (log *ε*) 204 (3.85), 256 (3.75), 293 (2.85), and 393 (2.65) nm; IR *ν*_max_ 3349, 3098, 2908, 1681, 1636, 1520, 1418, 1392, 1350, 1290, 1204, 1173, 1129, 1102, 1058, 1033, 932, 827, 803, 721, 667, and 609 cm^−1^; ^1^H NMR (MeOH-*d*_4_, 600 MHz) and ^13^C NMR (MeOH-*d*_4_, 150 MHz) spectral data, see Table 1; HRESIMS *m/z* 370.1299 [M + H]^+^ (calcd for C_20_H_20_NO_6_, 370.1285) and 739.2518 [2M + H]^+^ (calcd. for C_40_H_39_N_2_O_12_, 739.2498).

**TABLE 1:** NMR spectroscopic data for **1** ^a^.

No	1	
	*δ*_H_	*δ*_C_
1	8.34 s	132.3
3	4.87 t (6.6)	61.0
4	3.14 t (6.6)	28.4
4a		125.9
5		181.6
6	6.93 s	117.1
7		159.8
8		163.8
8a	8.08 s	156.8
9		136.8
10	2.78 s	16.3
1′		132.9
2′		124.7
3′		146.6
4′		152.7
5′	8.17 d (9.6)	127.3
6′	8.09 d (9.6)	121.3
7′	9.61 s	144.6
3′-OMe	4.15 s	62.6
4′-OMe	4.12 s	57.5

a NMR data (δ) were measured in MeOH-*d*_4_ for **1** at 600 MHz for ^1^H and at 150 MHz for ^13^C.

Proton coupling constants (J) in Hz are given in parentheses. The assignments were based on ^1^H–^1^H COSY, HSQC, HMBC, and NOESY experiments.

*Dehydrocorybulbine* (**2**): a yellow, amorphous powder; ^1^H NMR (DMSO-*d*_6_, 400 MHz) *δ*: 10.04 (1H, brs, 6-OH), 9.86 (1H, s, H-7′), 8.20 (1H, d, *J* = 9.6 Hz, H-5′), 8.16 (1H, d, *J* = 9.6 Hz, H-6′), 7.36 (1H, s, H-8), 6.91 (1H, s, H-5), 4.81 (2H, t, *J* = 5.6 Hz, H_2_-3), 4.09 (3H, s, 4′-OMe), 4.08 (3H, s, 3′-OMe), 3.86 (3H, s, 7-OMe), 3.05 (2H, t, *J* = 5.6 Hz, H_2_-4), and 2.97 (3H, s, Me-10); ^13^C NMR (DMSO-*d*_6_, 150 MHz) *δ*: 150.0 (C-4′), 149.1 (C-6), 146.3 (C-7), 143.9 (C-7′), 143.7 (C-3′), 136.4 (C-1), 133.2 (C-1′), 131.9 (C-4a), 129.3 (C-9), 125.9 (C-5′), 121.2 (C-6′), 120.6 (C-2′), 117.8 (C-8a), 115.1 (C-8), 114.6 (C-5), 62.0 (3′-OMe), 57.0 (4′-OMe), 56.8 (C-3), 56.2 (7-OMe), 26.6 (C-4), and 17.7 (Me-10) (+)-ESIMS *m/z* 352 [M]^+^.

### Bioassay for Anti-Cholinesterase Activity

Inhibition of AChE was assessed by a modified version of the colorimetric method of Ellman (Oh et al., 2004). A mixture of 196 μl phosphate buffer (PBS) and acetylcholinesterase (final concentration 0.01 *μ*g/ml) and 2 µl inhibitor (YH-D, compound **1**, and positive control donepezil) was added to the 96-well plate, and then the plate was incubated at 4°C for 20 min; 2 µl DTNB and ATCI (final concentration of 116 *μ*M) was added, and the solution was incubated for 20 min at 37°C on a shaker. The optical density was measured at 405 nm immediately, and the percentage inhibition was calculated. Donepezil was used as a positive control.

## Results and Discussion

The bioactive fraction YH-D (270 g) was separated systematically by column chromatography, such as MCI and Sephadex LH-20 as well as preparative HPLC, to yield secoyanhusamine A (**1**).

**FIGURE 2:**
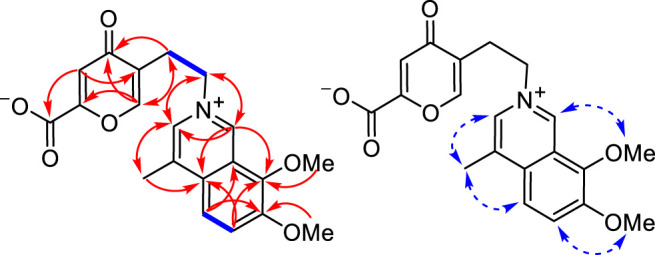
Main ^1^H–^1^H COSY (left, blue lines), three-bond HMBC (left, red arrows, from ^1^H to ^13^C), and NOESY (right, blue arrows) correlations of **1**.

Secoyanhusamine A (**1**) was obtained as a yellow amorphous powder. The HRESIMS of **1** gave the positive ion peak at *m/z* 370.1299 [M + H]^+^ (calcd. for C_20_H_20_NO_6_, 370.1285) and 739.2518 [2M + H]^+^ (calcd. for C_40_H_39_N_2_O_12_, 739.2498), corresponding to an elemental formula of C_20_H_19_NO_6_, which indicated 12 degrees of unsaturation. Its IR spectrum revealed the presence of an inner salt formed *via* carboxylate (3600-2800 and 1,636 cm^−1^), carbonyl (1,681 cm^−1^), and aromatic (1,520 and 1,418 cm^−1^) functionalities. The ^1^H NMR and ^1^H–^1^H COSY data (Figure 2) of **1** in MeOH-*d*_4_ provided the signals of two *ortho*-coupled protons at *δ*_H_ 8.17 (1H, d, *J* = 9.6 Hz, H-5′) and 8.09 (1H, d, *J* = 9.6 Hz, H-6′) and two nitrogen-bearing aromatic protons at *δ*_H_ 9.61 (1H, s, H-7′) and 8.34 (1H, s, H-1), which indicated a 4,7,8-trisubstituted isoquinoline moiety. Furthermore, two aromatic singlet protons at *δ*_H_ 8.08 (1H, s, H-8a) and 6.93 (1H, s, H-6), two adjacent coupling methylenes at *δ*_H_ 4.87 (2H, t, *J* = 6.6 Hz, H_2_-3) and 3.14 (2H, t, *J* = 6.6 Hz, H_2_-4), two methoxy groups at *δ*_H_ 4.15 (3H, s, OMe-3′) and 4.12 (3H, s, OMe-4′), and a methyl singlet at *δ*_H_ 2.78 (3H, s, H_3_-10) could be recognized in the ^1^H NMR spectrum of **1**. The ^13^C NMR data showed 20 carbon resonances corresponding to thirteen aromatic and/or olefinic carbons, two methylenes [*δ*_C_ 61.0 (C-3) and 28.4 (C-4)], two methoxy groups [(*δ*_C_ 62.6 (OMe-3′) and 57.5 (OMe-4′)], and a methyl group (*δ*_C_ 16.3, C-10) as well as two carbonyl carbons [(*δ*_C_ 181.6 (C-5) and 163.8 (C-8)] (Table 1). Since the above functionalities meet 11 degrees of unsaturation, compound **1** was considered as a highly oxidized isoquinoline alkaloid with another ring system.

The analysis of ^1^H–^1^H COSY, HSQC, HMBC, and NOESY NMR data confirmed the above assignments and constructed the structure of **1**. The proton resonances and corresponding proton-bearing carbon resonances in the NMR spectra were assigned by the HSQC experiment. The presence of the 4-methyl-7,8-dimethoxy-isoquinoline moiety of **1** was confirmed by the HMBC correlations from H-7′ to C-1′ and C-3′, from H-5′ to C-1′ and C-3′, from H-6′ to C-2′ and C-4′, from H_3_-10 to C-1′ and C-1, and from the two methoxy protons to C-3′ and C-4′ as well as the NOESY correlations of H_3_-10/H-1, OMe-4′/H-5′, and OMe-3′/H-7′ (Figure 2). Additionally, the ^1^H–^1^H COSY correlations of H_2_-3/H_2_-4 coupled with the HMBC cross-peaks from H_2_-4 to C-5 and C-8a, from H-6 to C-4a and C-8, and from H-8a to C-5 and C-7 served to establish a 5-(2-azanylethyl)-2-carboxylate-4-oxo-4*H*-pyran ring system (Figure 2). Finally, the observed HMBC cross-peaks from both H-1 and H-7′ to C-3 and from H_2_-3 to C-1 and C-7′ verified that the 5-(2-azanylethyl)-2-carboxylate-4-oxo-4*H*-pyran ring was connected to the 4-methyl-7,8-dimethoxy-isoquinoline moiety *via* the quaternary nitrogen atom (Figure 2). Therefore, the structure of **1** was determined as shown and named secoyanhusamine A. Taking into account the elemental composition and the presence of both alkali amine and acidic carboxylic units, **1** was considered to be an inner salt under a neutral hydrophilic condition. In a consistent manner, no TFA signals were observed in the ^13^C NMR and ^19^F NMR spectra of **1** (Supplementary Material, Figures 2, 3) when the purification of **1** was performed by semipreparative HPLC with TFA.

**FIGURE 3:**
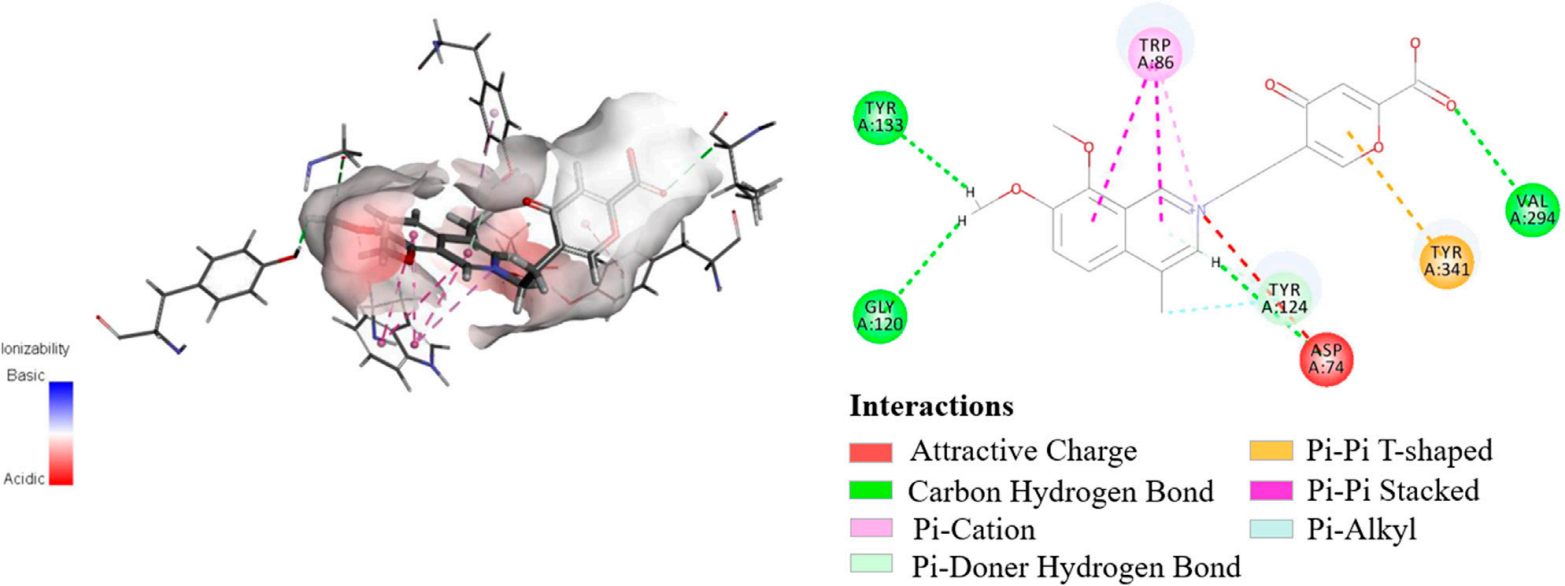
Molecular simulation docking results of **1** with AChE.

**SCHEME 1:**
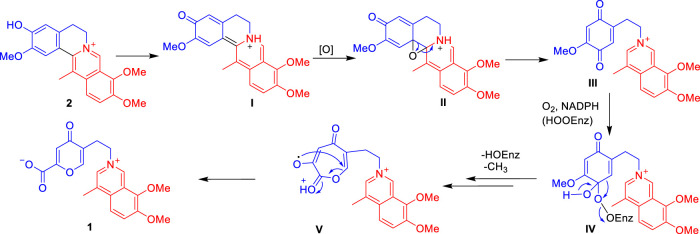
Proposed biogenetic pathways of **1**.

Isoquinoline alkaloids are the major constituents of *C. yanhusuo* (Hu et al., 2009; Zhou et al., 2012; Lv et al., 2012; Tong et al., 2013; Yang et al., 2014; Wang et al., 2020; Xiao et al., 2020). The 5-(2-azanylethyl)-2-carboxylate-4-oxo-4*H*-pyran ring moiety was not common in the isoquinoline alkaloids family. However, the remaining 4-methyl-7,8-dimethoxy-isoquinoline moiety of **1** was similar to that of isoquinoline alkaloids isolated from this genus plants (Lv et al., 2012; Tong et al., 2013; Yang et al., 2014; Liu et al., 2016; Wang et al., 2020; Xiao et al., 2020). Thus, **1** is probably generated from the biosynthetic precursor dehydrocorybulbine (**2**) (250 mg) (Lv et al., 2012), which has been also isolated from this plant in large quantities (Scheme 1). **2** undergoes an electron migration rearrangement followed by oxidative cleavage of ring B to obtain **III**, which continued to involve O_2_ and NADPH then cytochrome P450- or FAD-dependent enzymatic Baeyer–Villiger oxidation to form a peroxy-enzyme-activated complex **IV** (Liao et al., 2005; Ji et al., 2020; Fang et al., 2021), leading to the insertion of oxygen between C-8 and C-8a (**V**). The intermediate **V** would then undergo rearrangement to produce **1**.

The initial biological screening of the YH-D fraction encouraged further AChE inhibitory evaluation of secoyanhusamine A (**1**). As a result, secoyanhusamine A (**1**) showed potent inhibition with an IC_50_ value of 0.81 ± 0.13 *μ*M, compared to the positive control (donepezil, IC_50_ = 0.15 ± 0.01 *μ*M). Furthermore, molecular docking simulation was performed on the basis of the previously reported crystal structure of AChE (Cheung et al., 2012). As shown in Figure 3, secoyanhusamine A (**1**) could be docked perfectively into the catalytic cavity of AChE, while the *N*-atom and the pyrone ring could strongly interact with the Asp-74 residue of AChE *via* attractive charge and with the Tyr-341 residue of AChE *via* Pi–Pi T-shaped, respectively.

## Conclusion

In summary, secoyanhusamine A (**1**), a rare rearranged *seco*-isoquinoline alkaloid derived from ring oxidative cleavage, was isolated from an aqueous extract of *Corydalis yanhusuo* tubers and showed significant inhibitory activity against acetylcholinesterase (AChE). The structures of **1** were determined *via* extensive NMR spectroscopic analysis. Our study provides a new structural architecture of the natural product that can be used in follow-up studies relevant to the development of anti-Alzheimer’s agents.

## Data Availability

The original contributions presented in the study are included in the article/Supplementary Material, further inquiries can be directed to the corresponding authors.
