# Supplementary material for: Secoyanhusamine A, an Oxidatively Ring-Opened Isoquinoline Inner Salt From Corydalis yanhusuo
Source: Front Chem. 2022 Feb 1;9:831173. doi: 10.3389/fchem.2021.831173 (PMC8843934; doi:10.3389/fchem.2021.831173)

Supplementary Material

Secoyanhusamine A, an oxidatively ring opened isoquinoline inner salt from *Corydalis yanhusuo*

Lingyan Wang[1,2], Huan Xia[1], Yuzhuo Wu[1], Yanan Wang[2], Pengcheng Lin[3,*], Sheng Lin[1,*]

[1]Key Laboratory of Chinese Internal Medicine of Ministry of Education and Beijing, Dongzhimen Hospital, Beijing University of Chinese Medicine, Beijing 100700, People's Republic of China

[2]State Key Laboratory of Bioactive Substance and Function of Natural Medicines, Institute of Materia Medica, Chinese Academy of Medical Sciences and Peking Union Medical College, Beijing 100050, People's Republic of China

[3]College of Pharmaceutical Sciences, Qinghai University for Nationalities, Xining 810000, People's Republic of China

*** Correspondence:**
Corresponding Author
qhlpc@126.com (P.-C. Lin); lsznn@bucm.edu.cn (S. Lin)

Contents

Supplementary Figure 1. The ^{1}H NMR spectrum of compound **1** in MeOH-d_4 (600 MHz) 3

Supplementary Figure 2. The ^{13}C NMR spectrum of compound **1** in MeOH-d_4 (150 MHz) 4

Supplementary Figure 3. The ^{19}F NMR spectrum of compound **1** in MeOH-d_4 (400 MHz) 5

Supplementary Figure 4. The ^{1}H-^{1}H COSY spectrum of compound **1** in MeOH-d_4 (600 MHz)........6

Supplementary Figure 5. The HSQC spectrum of compound **1** in MeOH-d_4 (600 MHz) 7

Supplementary Figure 6. The HMBC spectrum of compound **1** in MeOH-d_4 (600 MHz) 8

Supplementary Figure 7. The NOESY spectrum of compound **1** in MeOH-d_4 (600 MHz)...............9

Supplementary Figure 8. The IR spectrum of compound **1** 10

Supplementary Figure 9. The HRMS spectrum of compound **1** in MeOH.......................................11

Supplementary Figure 10. The UV spectrum of compound **1** in MeOH 11

Supplementary Figure 11. Bioassay for anti-cholinesterase activity..12

Molecular docking studies…………………………………………………………………..12

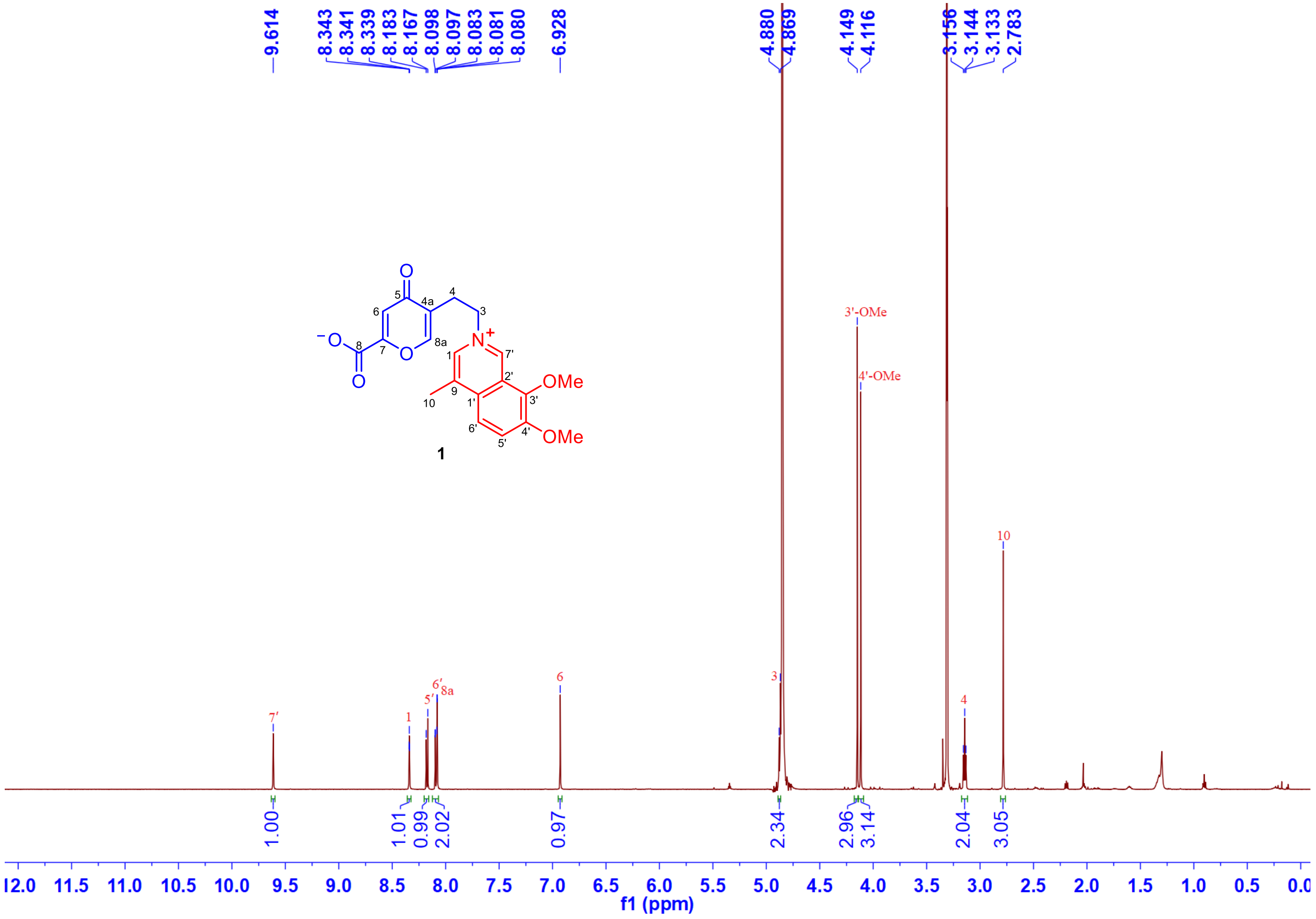

Supplementary Figure 1. The ^{1}H NMR spectrum of compound **1** in MeOH-d_4 (600 MHz)

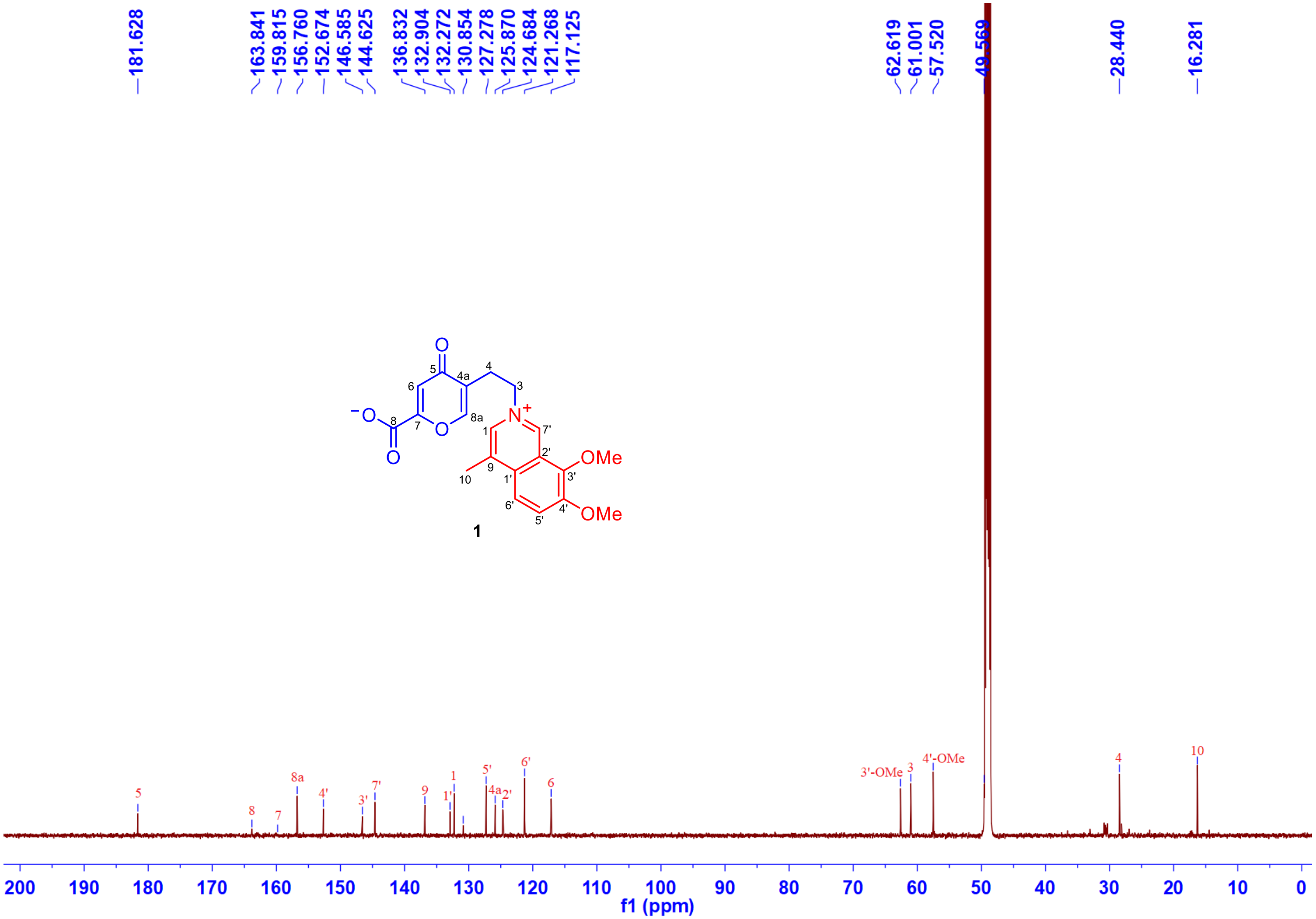

Supplementary Figure 2. The ^{13}C NMR spectrum of compound **1** in MeOH-d_4 (150 MHz)

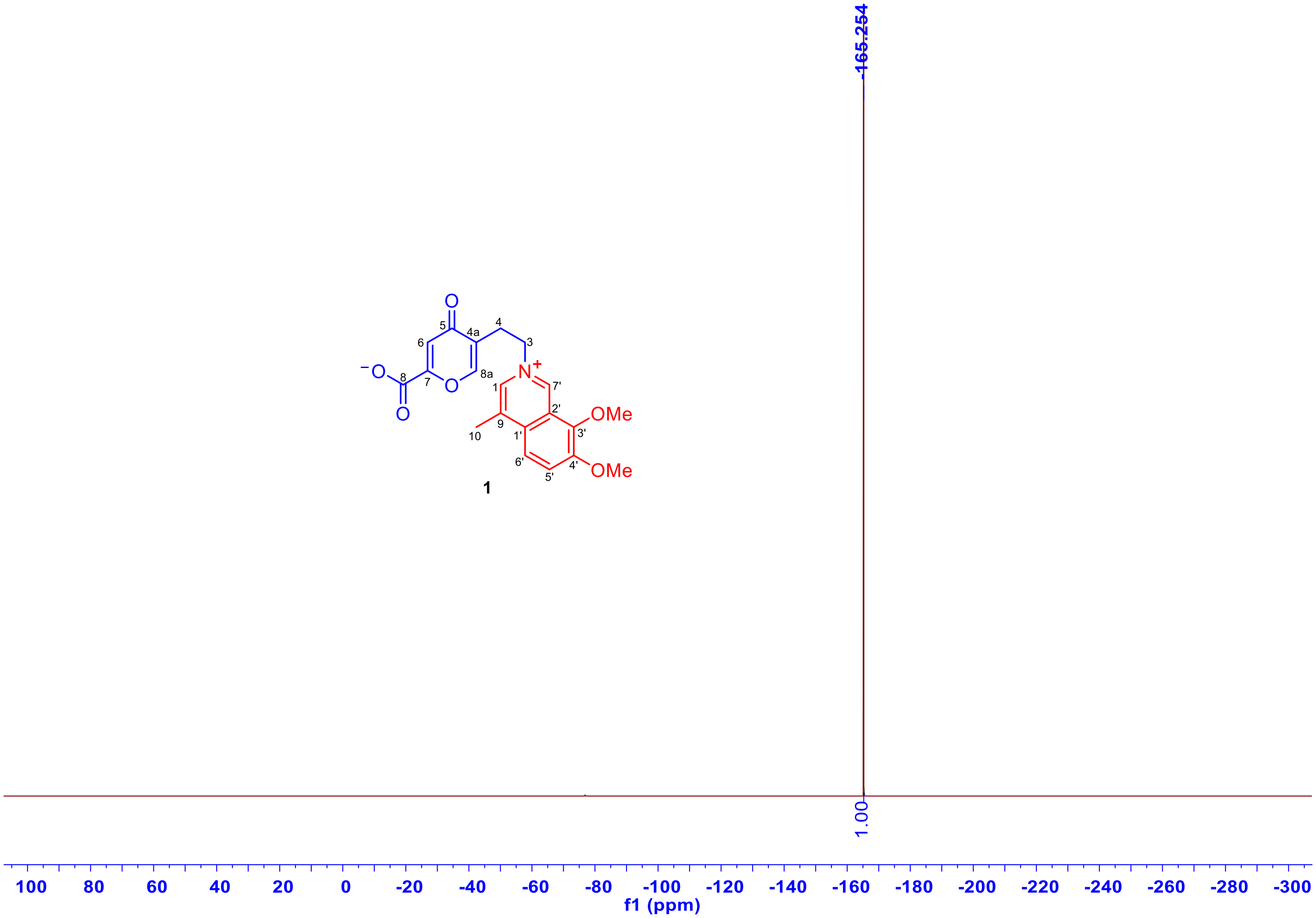

Supplementary Figure 3. The ^{19}F NMR spectrum of compound **1** in MeOH-d_4 (400 MHz)

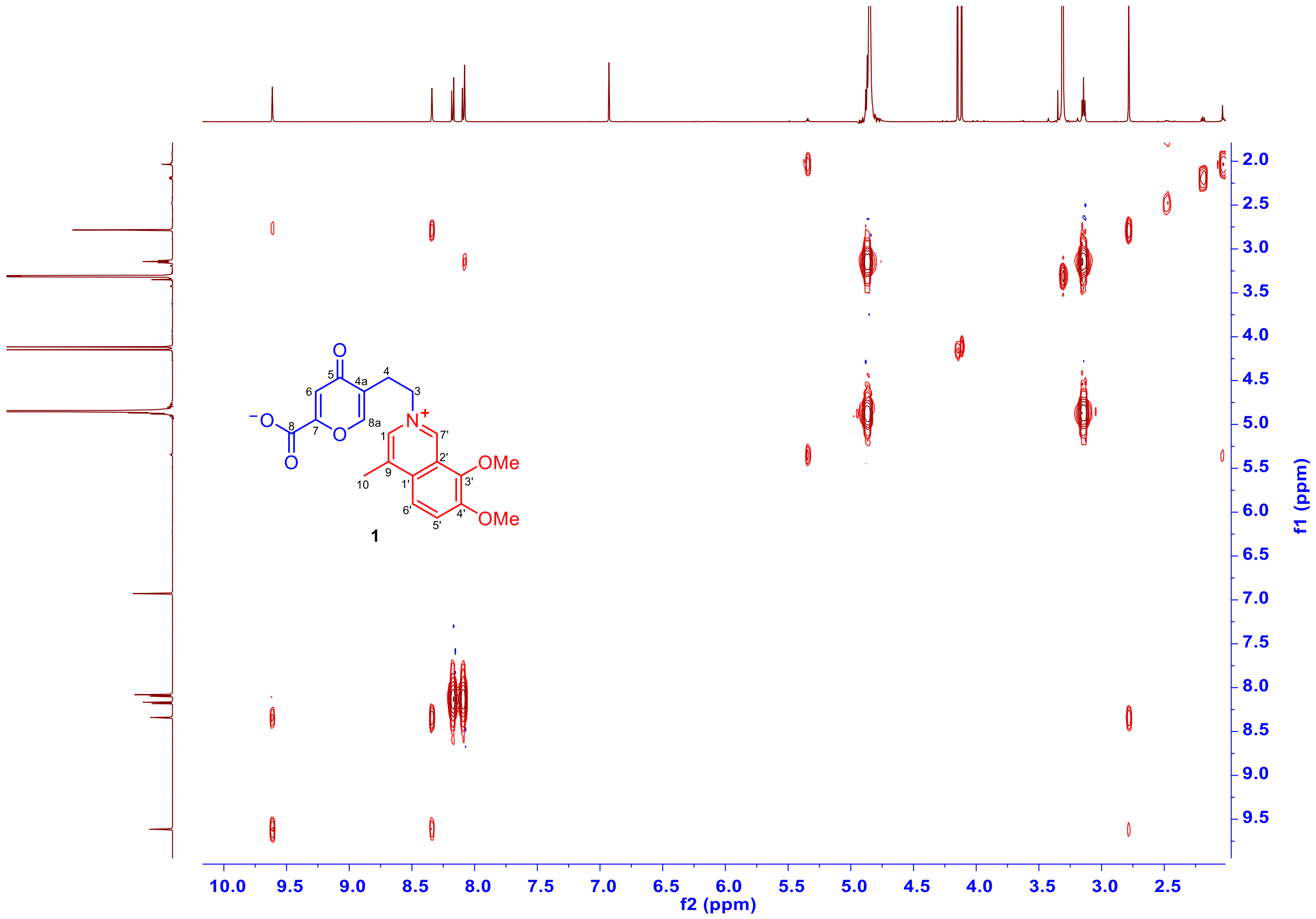

Supplementary Figure 4. The ^1H-^1H COSY spectrum of compound **1** in MeOH-d_4 (600 MHz)

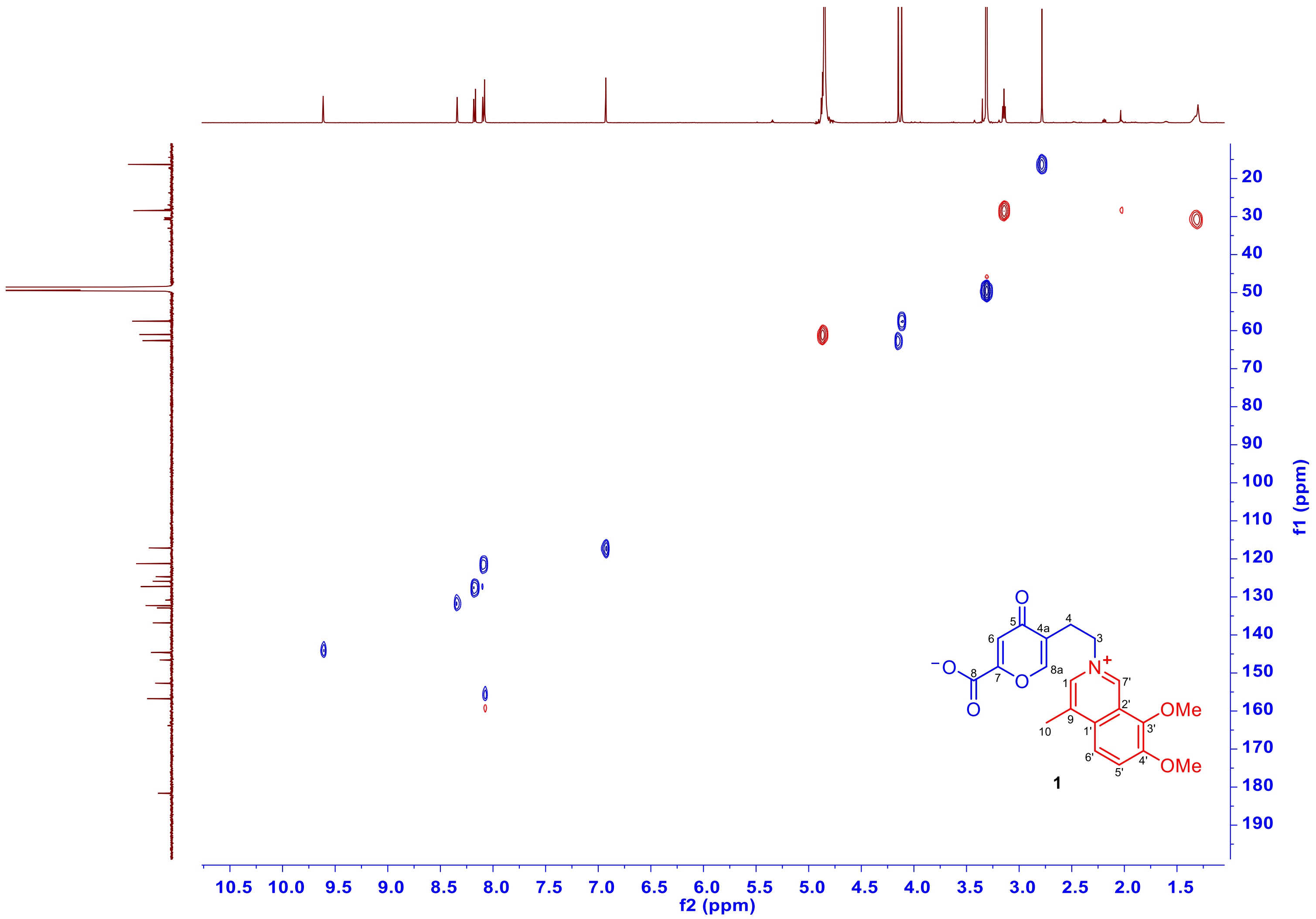

Supplementary Figure 5. The HSQC spectrum of compound **1** in MeOH-d_4 (600 MHz)

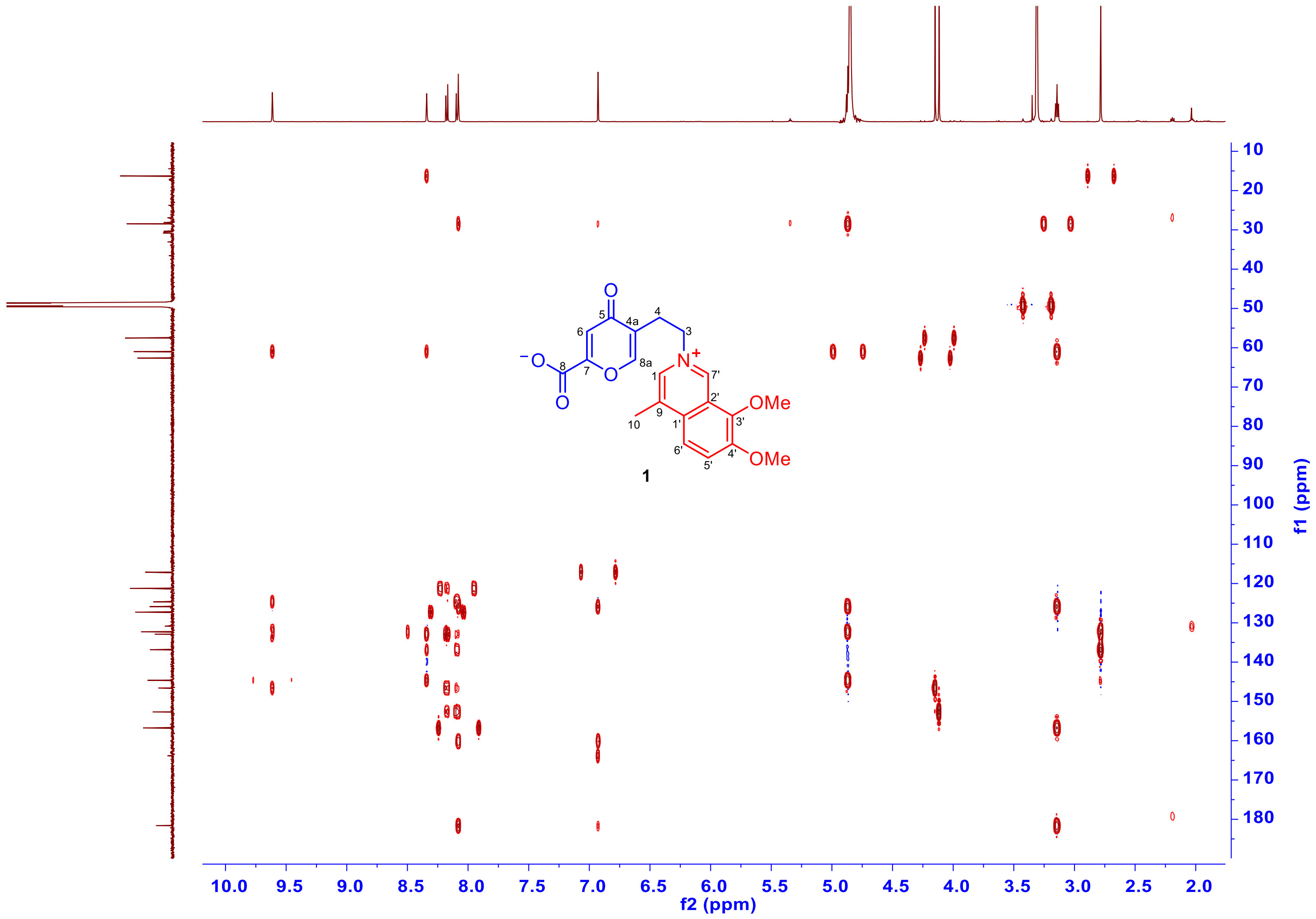

Supplementary Figure 6. The HMBC spectrum of compound **1** in MeOH-d_4 (600 MHz)

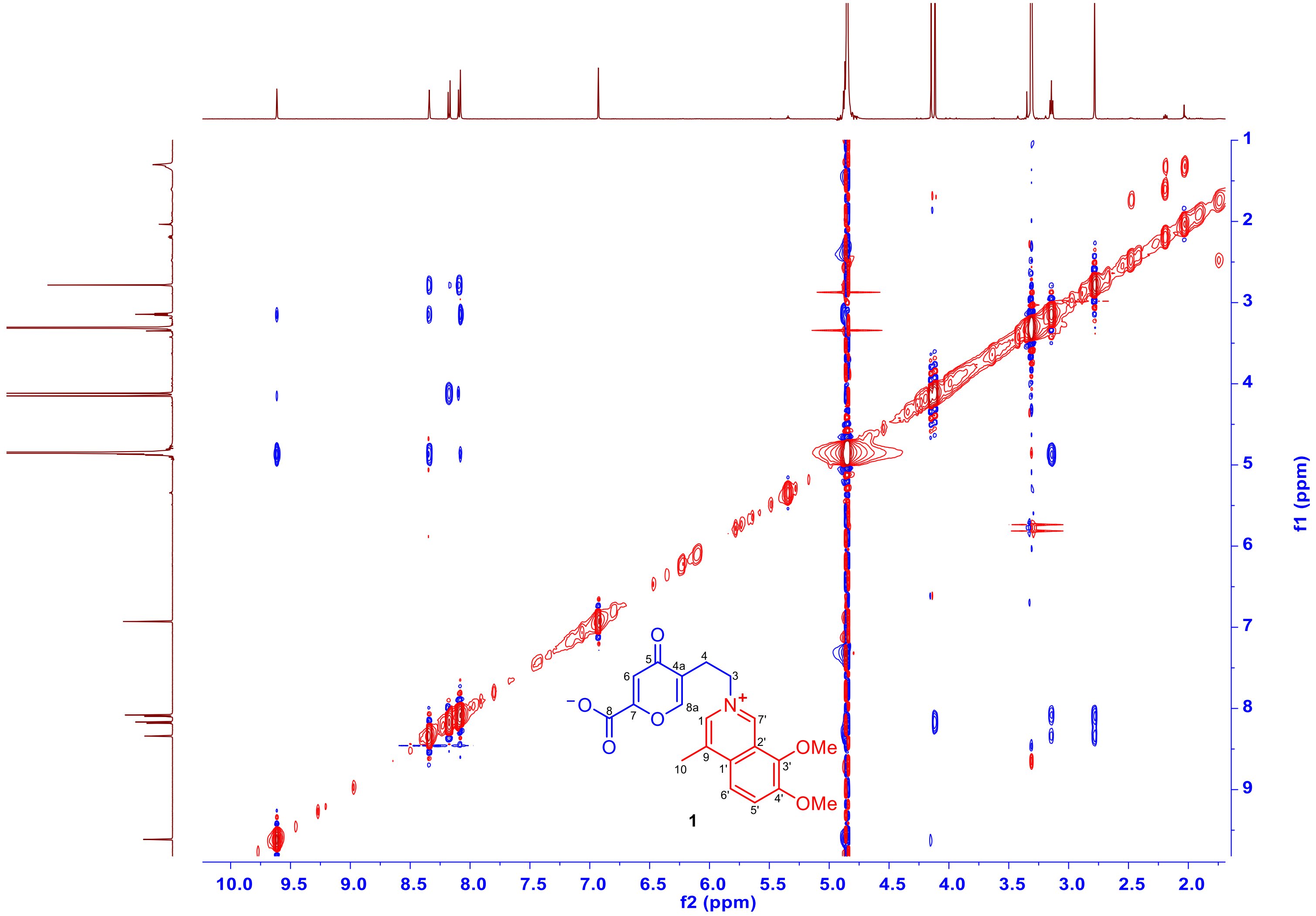

Supplementary Figure 7. The NOESY spectrum of compound **1** in MeOH-d_4 (600 MHz)

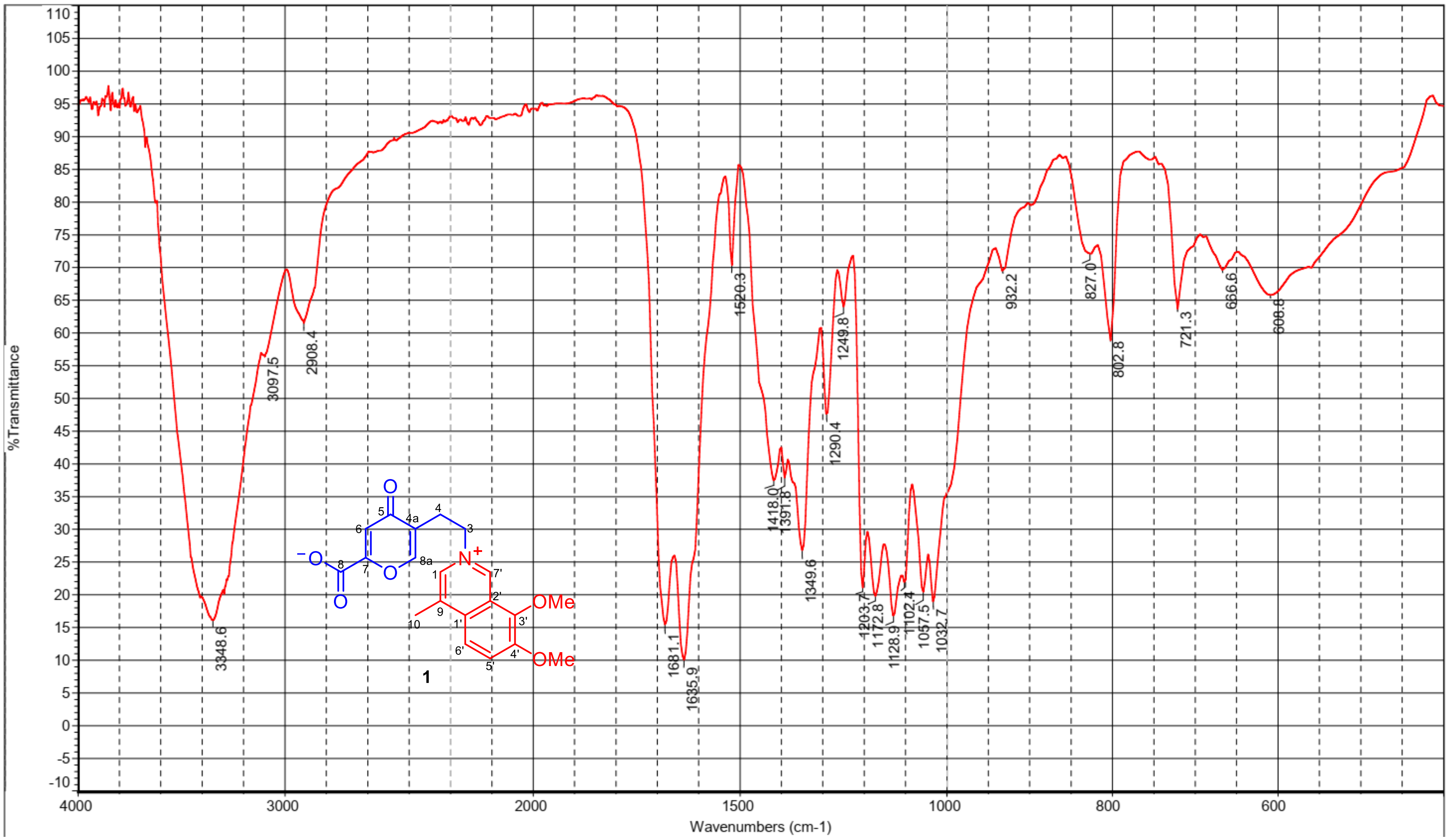

Supplementary Figure 8. The IR spectrum of compound **1**

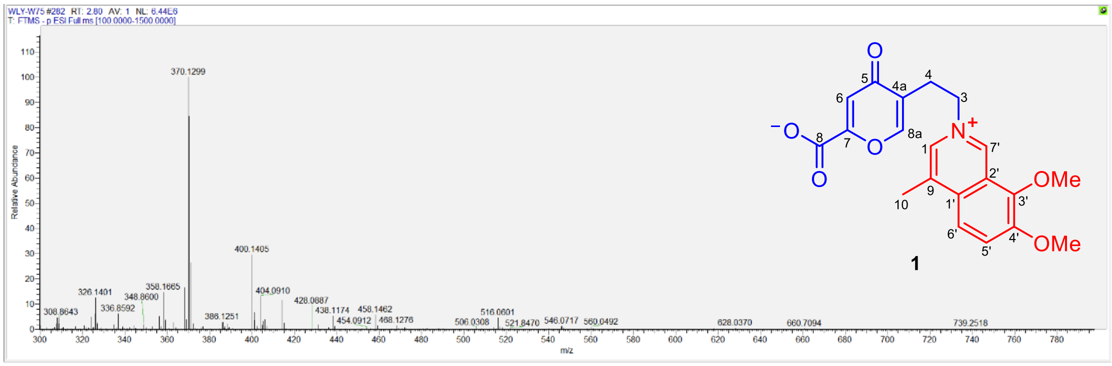

m/z	Theo. Mass	Delta (ppm)	RDB equiv.	Composition	
370.1299	370.1285	3.69	11.5	C20 H20 O6 N	M+H
739.2518	739.2498	2.81	22.5	C40 H39 O12 N2	2M+H

Supplementary Figure 9. The HRMS spectrum of compound **1** in MeOH

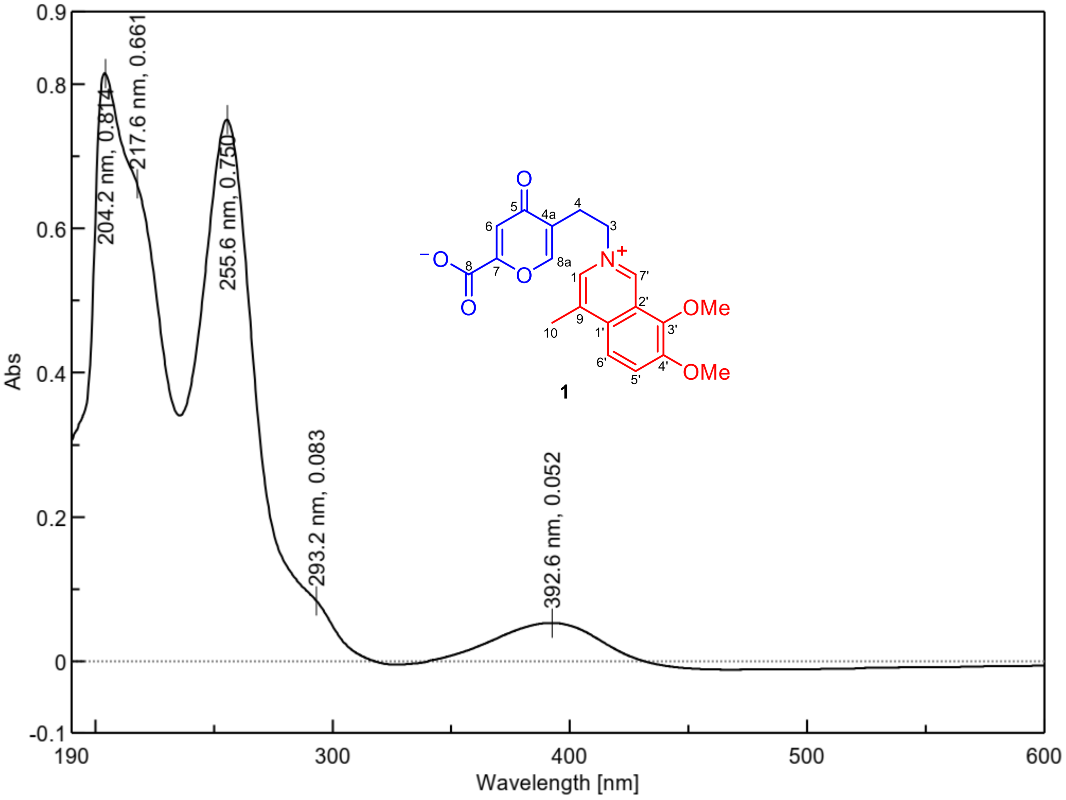

Supplementary Figure 10. The UV spectrum of compound **1** in MeOH

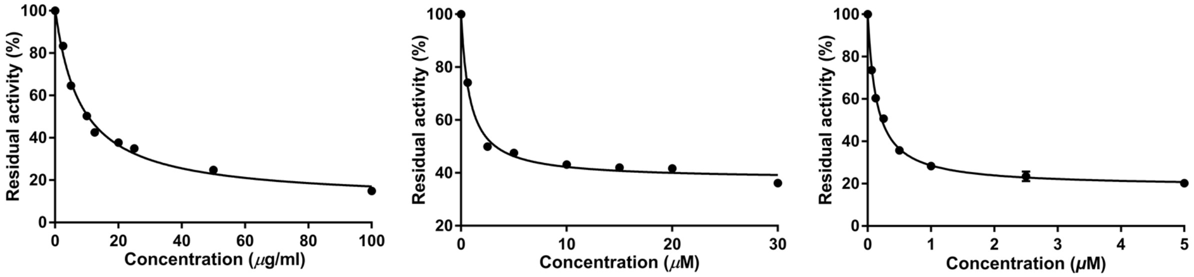

YH-D Compound **1** Donepezil

Supplementary Figure 11. Bioassay for anti-cholinesterase activity

Molecular docking studies

Molecular docking analyses in this study were performed using the software Discovery Studio 2016. The structural coordinates of AChE was based on the crystal structure of AChE, which was acquired from the PDB database (PDB: 4EY7). The protein and ligands were prepared by a standard procedure using Discovery Studio 2016. The location and the size of the active pocket was confined by the native ligand of AChE. The docking was performed using the "CDOCKER" procedure of Discovery Studio 2016.

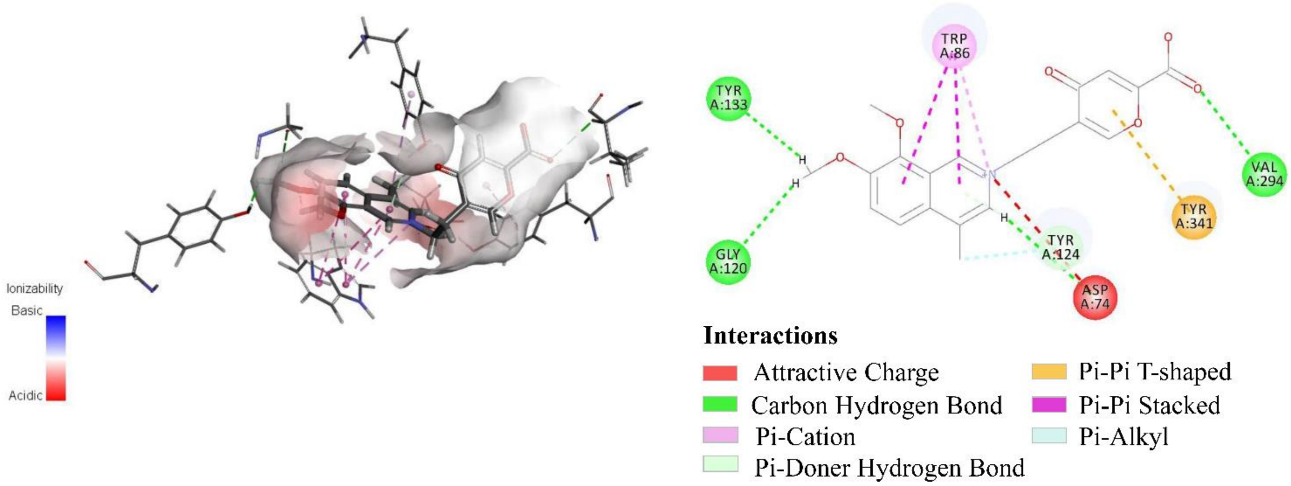

Supplement: Supplementary file 1 [file DataSheet1.pdf]
